# Psychological Capital and Work Engagement Among Mental Health Nurses in Saudi Arabia: The Mediation Role of Motivation at Work

**DOI:** 10.1155/jonm/6938783

**Published:** 2026-04-28

**Authors:** Samirh Said Alqhtani, Nouf Afit Aldhafeeri, Abdulraheem Mulfi Almutairy, Arwa Alsharekh, Sarah Alanazi, Laila Al Soraia, Aryam Suhail Alotaibi, Norah Alharbi

**Affiliations:** ^1^ College of Nursing, King Saud Bin Abdulaziz University for Health Sciences, Riyadh, Saudi Arabia, ksau-hs.edu.sa; ^2^ King Abdullah International Medical Research Center, Riyadh, Saudi Arabia, kaimrc.med.sa; ^3^ Ministry of the National Guard-Health Affairs, Riyadh, Saudi Arabia; ^4^ Eradah and Mental Health Complex-Mental Services, Ministry of Health, Jeddah, Saudi Arabia, moh.gov.sa

**Keywords:** mental health nurse, psychological capital and work engagement, Saudi Arabia, work motivation

## Abstract

**Background:**

Mental health nurses (MHNs) often work in challenging work environments that can affect their emotional well‐being and job performance. The high work demands, stress, and limited resources could influence MHNs’ psychological capital (PsyCap), motivation, and engagement. Therefore, this study aims to explore the relationship between PsyCap and work engagement (WE), with a focus on the mediating role of motivation at work (MAW) among MHNs.

**Methods:**

This study used a descriptive multivariate correlational design. A convenience sample was used to recruit 210 MHNs from a psychiatric hospital in Saudi Arabia in western region. MHNs completed three validated online questionnaires: the Psychological Capital Questionnaire–Short Version (PCQ‐12), the Utrecht Work Engagement Scale (UWES‐9), and the Motivation at Work Scale (MAWS). Data were analyzed using R software, performing descriptive and inferential tests and structural equation modeling (SEM).

**Results:**

PsyCap showed a positive relationship with MAW (*r* = 0.67, *p* < 0.001) and WE (*r* = 0.67, *p* < 0.001). MAW was strongly associated with WE (*r* = 0.87, *p* < 0.001). SEM revealed that the influence of PsyCap on WE was fully mediated by MAW (direct path ß = 0.00. *p* = 0.938; indirect *ß* = 0.74, *p* < 0.001). The result showed that professional title predicts MAW and WE; age and educational level were predictors of PsyCap.

**Conclusion:**

PsyCap contributes to higher levels of WE among MHNs, with MAW playing a central mediating role. These findings highlight the importance of motivation in enabling psychological resources to be translated into WE. Therefore, targeted interventions that focus on enhancing MAW may improve WE, enhance nurse retention, and improve patient outcomes.

## 1. Background

Mental health nurses (MHNs) frequently work in highly demanding settings involving workload shifts, rotating, prolonged working hours, and limited resources [1]. MHNs are also exposed to emotional stressors, interpersonal conflicts, and psychological threats, which can affect their performance and social relationships [2]. Therefore, maintaining a positive psychological state is important to enhancing resilience, achieving goals, and enhancing performance [3].

Psychological capital (PsyCap) is defined as the positive psychological development of an individual, including self‐efficacy, optimism, hope, and resilience [4]. Optimistic nurses tend to maintain high levels of motivation and job satisfaction, while nurses with high levels of hope set achievable goals and sustain strong work motivation [5]. Further, resilience enables nurses to adopt positive attitudes and manage stress effectively [6]. PsyCap facilitates adaptive coping with stress and workplace demands, thus promoting engagement and optimal professional functioning. Previous studies suggest that PsyCap contributes to reducing job stress and burnout among MHNs [3], enhances job satisfaction, commitment, and well‐being, and improves patient outcomes [7, 8].

Work engagement (WE) is a multidimensional concept explaining the psychological state of persons who are enthusiastic and absorbed in their work [9]. WE contributes to positive and professional outcomes, which are characterized by vigor, dedication, and absorption [9]. Recent studies showed that WE enhances MHNs’ mental well‐being, job attitudes, and quality of care [10]. Further, multiple factors influence WE such as demographics, workplace conditions, individual traits, and patient‐related factors [11]. WE increases job satisfaction and organizational commitment and improves emotional well‐being [12]. Further, a supportive supervisor and organizational culture that provide autonomy can foster WE through perceived organizational support and increased motivation [11, 13]. In addition, strategies such as job enrichment, peer support, and participatory decision‐making processes can alleviate burnout and enhance WE through a sense of belonging [14, 15].

Motivation is a fundamental determinant of human behavior. Motivation at work (MAW) consists of internal and external drives that compel individuals to act, perform, and achieve their goals [16, 17]. Both motivational factors have been shown to significantly influence individual behavior and outcomes [18]. Intrinsic motivation arises from internal satisfaction, achievement, and self‐esteem, whereas extrinsic motivation is shaped by external rewards such as income, job satisfaction, job characteristics, and the work environment [19]. In the nursing field, motivation is essential for promoting quality of care, ensuring patient safety, and supporting patient‐centered practice [20]. Further, MAW enhances self‐reliance, personal growth, and achievements among nurses [20].

Evidence showed that PsyCap positively influences WE by strengthening commitment and performance [21]. WE is influenced by both organizational and individual factors, including a supportive work environment and strong psychological resources [22]. Consequently, fostering a positive organizational atmosphere and strengthening PsyCap among nurses may enhance WE and satisfaction [22, 23]. In contrast, evidence showed that low PsyCap reduces WE across varied professional settings [24]. PsyCap has a vital role in enhancing MAW, as intrinsic motivation and goal commitment can mediate the relationship between PsyCap and job performance, thus strengthening WE [23]. Previous studies support the positive relationship between MAW and WE, which improves the quality of care among Jordanian nurses [25] and among MHNs in Japan’s long‐term facility [11].

Although previous studies have examined the relationship between PsyCap and WE, as well as that between WE and MAW, the mediating role of MAW in the link between PsyCap and WE among MHNs remains unexplored, especially within the Saudi healthcare context. Exploring this relationship is important for enhancing PsyCap and WE to achieve optimal patient outcomes in mental health settings. Healthcare institutions can improve nurse performance and quality of care by strengthening nurses’ motivation, which in turn fosters PsyCap and WE. Nurse managers and administrators can use motivation‐focused strategies to enhance PsyCap and WE. Based on theoretical perspectives and previous research evidence, this study proposes that PsyCap (independent variable) enhances MAW (mediating variable), which in turn increases WE (dependent variable).

### 1.1. Theoretical Framework

Job demand‐resources (JD‐R) theory [26] and self‐determination theory (SDT) [27] were used as interpretive lenses that formulate the conceptual model of this study. JD‐R theory proposes that job resources and personal resources have more potential to increase WE and job performance. Job resources include physical, social, or organizational elements of the work that help employees in meeting job goals and supporting their professional development [26]. These resources contribute to a motivational pathway that enhances WE and leads to positive outcomes. In JD‐R theory, personal resources, particularly PsyCap involving self‐efficacy, optimism, hope, and resilience, serve as important precursors to WE.

**FIGURE 1:**
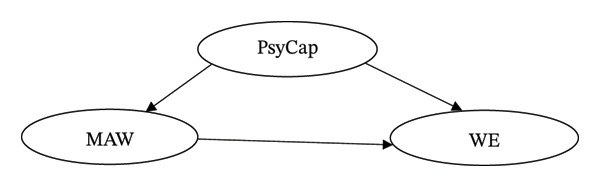
Conceptual framework between PsyCap, MAW, and WE.

According to SDT, both intrinsic and extrinsic motivational factors are influenced by personal needs and environments factors [27]. The satisfaction of three basic psychological needs (autonomy, competence, and relatedness) is essential for fostering motivation and WE. In the current study, it is proposed that when MHNs perceive their work environments as supportive of these psychological needs, they will develop their personal resources such as PsyCap and experience internal motivation that maintains their engagement. By integrating both JD‐R and SDT in this study, personal resources (PsyCap) enhanced motivation that fosters positive outcomes (WE). Thus, MAW is a mediating pathway. The conceptual framework in Figure 1 presents the expected relationships between study variables. The following were the hypotheses proposed in this study:

 H1PsyCap is positively associated with WE among MHNs
 H2PsyCap is positively associated with MAW among MHNs
 H3MAW is positively associated with WE among MHNs
 H4: MAW mediates the relationship between PsyCap and WE among MHNs

## 2. Aim of Study

This study aims to examine the relationship between MAW, WE, and PsyCap and the mediating role of MAW among MHNs.

## 3. Methodology

### 3.1. Design and Participants

A descriptive cross‐sectional correlational design was used to examine the relationship between the study variables, generate hypotheses, and as a cost‐effective approach [28]. Participants were recruited using a convenience sampling technique; participants who met the study inclusion criteria, were available, and willing to participate in this study were included [29]. This study was conducted in a mental health hospital located in the western region of Saudi Arabia. The inclusion criteria were MHNs with at least 1 year of experience at the hospital and consented to participate. The exclusion criteria were nurses who were students, interns, or nurses working in nonpsychiatric units.

### 3.2. Sample Size

Monte Carlo simulations were conducted using the lavaan package in R software to estimate a sample size for structural equation modeling (SEM). The population model involved three latent constructs, PsyCap, WE, and MAW, with MAW serving as a predictor of both PsyCap and WE, and also serving as a predictor of WE. WE was regressed on 17 sociodemographic covariates. Simulated databases were generated for samples ranging from 100 to 1000, with 100 replications per condition. Model fit was evaluated using standard fit indices, root mean square error of approximation (RMSEA), Comparative Fit Index (CFI), Tucker–Lewis Index (TLI), and standardized root mean square residual (SRMR).

The simulation results indicated that a sample of 200 achieved an acceptable model fit.

The following values met the recommended thresholds for good model fit: RMSEA = 0.0165, DFI = 0.973, TLI = 0.075, and SRMR = 0.047. These values indicate that a sample of 200 participants is sufficient for robust parameter estimation and hypothesis testing. In this study, the final sample size was 210 participants, where three participants withdrew from the study. Demographic data and professional covariates were chosen a priori to account for confounding effects, which resulted in 22 predictors after indicator coding included in the regression model. A power analysis for multiple regression was performed using the pwr.f2.test function (R package) with *u* = 22, *f* ^2^ = 0.15, α = 0.05, and power = 0.80. The minimum required sample was 163, thus supporting the adequacy of the final sample and inclusion of all covariates.

### 3.3. Data Collection

Following the institutional review board (IRB) approval, a Microsoft Forms survey link was emailed to the head of the nursing department at a psychiatric and mental health hospital. The study was carried out in a large specialized psychiatric facility in the western region of Saudi Arabia. This hospital provides a wide range of services, including outpatient clinics, inpatient units, addiction treatment, and home healthcare services, which was considered a suitable setting for this study.

Data were collected throughout 3 months, from January to March 2025. The online survey included a cover page with the study’s purpose, a consent form, followed by demographic data and validated tools. The estimated time to complete the survey was 8–10 min. Using the online survey minimized potential bias by not collecting any participants’ identifying information and by allowing participants to complete the survey privately and independently. Duplication of filling the survey was prevented by allowing each participant to submit only one response.

#### 3.3.1. Measurements

A. Demographic data: Participants’ demographic data included the following: gender, marital status, age, level of education, years of experience, and professional title.
B. Motivation at Work Scale (MAWS): The MAWS consisted of 12 items with a 7‐point Likert scale. The rating of this scale varies between 1 and 7 points, with the option “*Strongly Disagree*” assigned a score of 1 and “*Strongly Agree*” assigned a score of 7. The tool has four key domains of MAW: external regulation, introjection, identification, and intrinsic motivation [30]. The total score reflected the level of MAW, with higher scores indicating more motivation. Internal consistency for the scale was 0.95, and subscales ranged from 0.92–0.95 [25]. The MAWS has been cross‐culturally validated by experts in 10 different languages, making it a tool that can be used in several countries [30]. In this study, MAWS’ Cronbach’s alpha was 0.92 (95% confidence interval [CI]: 0.90–0.94), indicating excellent internal consistency.
C. Utrecht Work Engagement Scale (UWES‐9): The UWES‐9 is a self‐report scale used to measure WE [31]. The UWES consisted of nine items on a 7‐point Likert scale, where higher scores indicate greater levels of engagement. This tool has three subscales that involves vigor, dedication, and absorption. The rating scores varied from 0 (*Never*) to 6 (*Always every day*), with total score ranging from 9 to 45. The higher score indicates a greater level of engagement at work. Nurses feel energized (vigor), enthusiastic and proud (dedicated), and fully immersed (absorption) in their job. The UWES has shown strong psychometric properties across different populations. For instance, the Greek version of the UWES demonstrated high reliability with a Cronbach’s alpha of 0.924 and confirmed the three‐factor structure through confirmatory factor analysis [32]. Similarly, the UWES showed moderately high reliability in Zimbabwe [33]. Concurrent validity was reported, particularly in nursing [32]. For UWES, the internal consistency in this study was excellent, with a Cronbach’s alpha of 0.95 (95% CI: 0.94–0.97).
D. Psychological Capital Questionnaire–Short Version (PCQ‐12): The PCQ‐12 is a 6‐point Likert scale that is used to measure PsyCap. This scale ranges from 1 (*Strongly Disagree*) to 6 (*Strongly Agree*) [34]. The PsyCap tool includes four key concepts: efficacy, optimism, hope, and resilience. Each subscale score is determined by calculating the mean (average) of its 6 items, and the overall PsyCap score is obtained by averaging all 12 items. The overall score reflects an individual’s level of positive PsyCap, with a high score indicating higher PsyCap [35]. Additionally, an average of the items for each dimension was obtained to calculate the score as follows: efficacy: Items 1–3, hope: Items 4–7, resilience: Items 8–10, and optimism: Items 11–12. The reliability and validity of the tool were reported in various studies. The PCQ‐12 had good Cronbach’s alpha reported near 0.89 [36], with good convergent validity[37, 38]. In this study, PsyCap’s Cronbach’s alpha was 0.94 (95% CI: 0.92–0.96), suggesting excellent internal consistency. All tools’ permissions were obtained.

### 3.4. Ethical Consideration

In accordance with the Declaration of Helsinki, the study was approved by the Ethical Committee of King Abdullah International Medical Research Center (KAIMRC) (IRB# 00000100625, NRR24/015/12) and the Ministry of Health (IRB # A02118). An online digital consent form was obtained. The consent form covered the study’s purpose and informed participants that they could withdraw from the study at any time. Confidentiality and privacy were maintained during all study processes.

### 3.5. Data Management and Analysis Plan

R software (Version 4.2.2) was used in conducting all data analyses with a significance level set at *p* < 0.05. Descriptive statistics were used to summarize demographic and professional characteristics. Pearson correlations were used to assess the direction and strength of the relationship between all variables. Preliminary univariable analyses were used to examine the correlation between demographic variables and study outcomes. Multivariable linear regression analysis was performed to examine the independent effects of demographic and professional factors, reporting mean differences (MDs), 95% CIs, and *p* values.

The hypothesized mediation model in this study was tested using SEM via “lavaan” package that was used to test the measurement and structural models, with fit indices including *χ* ^2^/df, CFI, TLI, RMSEA, and SRMR. The SEM analyses provided standardized path coefficients to evaluate both direct and indirect effects within the model.

The mediation analyses were conducted using the Preacher–Hayes method via the “mediation: package in R to assess whether MAW mediated the relationship between PsyCap and WE. Bootstrapping with 1000 resamples was applied to estimate the indirect effects and 95% CIs.

## 4. Results

### 4.1. Demographics Data

Among 210 psychiatric nurses, 52.9% were female and 47.1% were male. Most participants were aged 30–39 (50.5%), 51.0% held a bachelor’s degree, and 25.7% had 6–10 years of experience. The majority were married (58.1%). The most common professional title was staff nurse (55.7%) (Table 1).

**TABLE 1:** Demographics and professional data of the study sample.

Variable	Frequency (%)
Age	
20–29	31 (14.8)
30–39	106 (50.5)
40–49	64 (30.5)
50 and above	9 (4.3)
Gender	
Male	99 (47.1)
Female	111 (52.9)
Level of education	
Diploma	83 (39.5)
Bachelor’s degree	107 (51.0)
Master’s degree	18 (8.6)
PhD	2 (1.0)
Years of experience	
1–5 years	44 (21.0)
6–10 years	54 (25.7)
11–15 years	49 (23.3)
16–20 years	39 (18.6)
More than 20 years	24 (11.4)
Marital status	
Married	122 (58.1)
Single	65 (31.0)
Divorced	23 (11.0)
Professional title	
Staff nurse	117 (55.7)
Charge nurse	50 (23.8)
Head nurse	19 (9.0)
Nursing supervisor	16 (7.6)
Nursing coordinator	4 (1.9)
Nursing manager	4 (1.9)

### 4.2. Levels of MAW, WE, and PsyCap Among MHNs

The overall MAWS was 4.8 ± 1.3, with subscale means ranging from 4.6 ± 1.6 for external regulation to 4.9 ± 1.6 for intrinsic motivation. For UWES, the mean total score was 4.9 ± 1.4, with the highest subscale mean for dedication (5.1 ± 1.4). PsyCap had a mean total score of 4.8 ± 0.9, with subscales ranging from 4.6 ± 1.0 for resilience to 4.9 ± 1.0 for efficacy, hope, and optimism (Table 2).

**TABLE 2:** Levels of work motivation, work engagement, and psychological capital among the study sample.

Scales	Mean ± SD
Motivation at work (MAWS)	
Intrinsic motivation	4.9 ± 1.6
Identification	4.8 ± 1.5
Introjection	4.8 ± 1.6
External regulation	4.6 ± 1.6
Total MAWS	4.8 ± 1.3
Work engagement (UWES)	
Vigor	4.8 ± 1.6
Dedication	5.1 ± 1.4
Absorption	4.8 ± 1.5
Total UWES	4.9 ± 1.4
Psychological capital (PsyCap)	
Efficacy	4.9 ± 1.0
Hope	4.9 ± 0.9
Resilience	4.6 ± 1.0
Optimism	4.9 ± 1.0
Total PsyCap	4.8 ± 0.9

### 4.3. Univariable Associations Between Demographic Data and MAW, WE, and PsyCap

Table 3 presents the univariable associations between demographic data and MAW, WE, and PsyCap total scores. Total MAW scores were significantly associated with age (*p* = 0.015) and professional title (*p* = 0.002). Nurses aged 40–49 years reported the highest mean MAW score (5.2 ± 1.5), while those aged 50 and above had the lowest (4.1 ± 1.3). Charge nurses had the highest mean MAW score (5.3 ± 1.4) compared to other titles. No significant associations were observed for gender, marital status, education level, or years of experience.

**TABLE 3:** Univariable associations between demographic data and total scores of MAWS, WE, and PsyCap.

Variable	Total MAW		Total WE		Total PsyCap	
	Mean ± SD	*p*	Mean ± SD	*p*	Mean ± SD	*p*
Age						
20–29	4.5 ± 1.3	0.015^∗^	4.7 ± 1.5	0.072	4.8 ± 0.8	0.171
30–39	4.7 ± 1.1		4.9 ± 1.3		4.9 ± 0.7	
40–49	5.2 ± 1.5		5.2 ± 1.6		4.9 ± 1.1	
50 and above	4.1 ± 1.3		4 ± 1.3		4.2 ± 1	
Gender						
Male	4.9 ± 1.2	0.209	5.1 ± 1.3	0.125	4.8 ± 0.9	0.815
Female	4.7 ± 1.4		4.8 ± 1.5		4.8 ± 0.9	
Marital status						
Married	4.7 ± 1.3	0.284	4.8 ± 1.5	0.358	4.8 ± 0.9	0.531
Single	5 ± 1.2		5.1 ± 1.3		4.9 ± 0.8	
Divorced	4.7 ± 1.1		4.8 ± 1.1		4.7 ± 0.9	
Level of education						
Diploma	5 ± 1.3	0.098	5.1 ± 1.4	0.131	4.9 ± 0.9	0.059
Bachelor’s degree	4.7 ± 1.2		4.8 ± 1.4		4.8 ± 0.8	
Master’s/PhD degrees	4.5 ± 1.6		4.6 ± 1.6		4.4 ± 1.3	
Years of experience						
1–5 years	4.4 ± 1.2	0.141	4.7 ± 1.6	0.497	4.6 ± 0.8	0.605
6–10 years	4.8 ± 1.1		4.9 ± 1.2		4.8 ± 0.8	
11–15 years	4.8 ± 1.2		5 ± 1.4		4.9 ± 0.7	
16–20 years	4.9 ± 1.5		4.9 ± 1.5		4.8 ± 1.1	
More than 20 years	5.3 ± 1.5		5.3 ± 1.5		5 ± 1	
Professional title						
Staff nurse	4.5 ± 1.1	0.002^∗^	4.7 ± 1.3	0.008^∗^	4.7 ± 0.8	0.314
Charge nurse	5.3 ± 1.4		5.5 ± 1.4		5 ± 1	
Head nurse	4.9 ± 1.5		5 ± 1.7		4.7 ± 1.2	
Nursing supervisor/coordinator/manager	4.8 ± 1.4		4.9 ± 1.4		5 ± 0.8	

*Note: p* values are for *t*‐tests in cases of binary factors and analysis of variance in cases of factors with more than two categories.

^∗^ *p* < 0.001.

For total WE scores, significant associations were found with professional titles (*p* = 0.008), with charge nurses reporting the highest mean score (5.5 ± 1.4). No significant associations were observed with age, gender, marital status, education level, or years of experience. In terms of the total PsyCap scores, there were no significant associations with demographic data.

### 4.4. The Multivariable Linear Regression Analyses of Demographic Data on the Total Scores of MAW, WE, and PsyCap

The results of the multivariable linear regression analyses examine the effect of demographic data on the total scores of MAW, WE, and PsyCap. For total MAW scores, being a charge nurse was significantly associated with higher scores (MD = 0.61, 95% CI: 0.16 to 1.06, *p* = 0.008). No significant associations were observed for age, gender, marital status, education level, or years of experience.

Regarding total WE scores, charge nurses also reported significantly higher scores (MD = 0.64, 95% CI: 0.14 to 1.13, *p* = 0.013). Other demographic and professional variables did not show significant associations.

For total PsyCap scores, being aged 50 and above was significantly associated with lower scores (MD = −0.93, 95% CI: −1.73 to −0.12, *p* = 0.025). Additionally, having a master’s or PhD degree was significantly associated with lower PsyCap scores (MD = −0.59, 95% CI: −1.06 to −0.12, *p* = 0.016). No other significant associations were found for gender, marital status, professional title, or years of experience (Table 4).

**TABLE 4:** Multivariable linear regression analyses for the effect of participants’ demographics/professional data on the total scores of MAW, WE, and PsyCap.

Factor	Total MAW		Total UWE		Total PsyCap	
	MD (95% CI)	*p*	MD (95% CI)	*p*	MD (95% CI)	*p*
(Intercept)	4.41 (3.67, 5.14)	< 0.001^∗^	4.91 (4.09, 5.72)	< 0.001^∗^	4.69 (4.18, 5.21)	< 0.001^∗^

*Age*						
20–29	Reference					
30–39	0.10 (−0.55, 0.76)	0.756	0.00 (−0.73, 0.72)	0.990	−0.06 (−0.52, 0.40)	0.804
40–49	0.30 (−0.46, 1.07)	0.436	0.12 (−0.73, 0.97)	0.786	−0.18 (−0.72, 0.36)	0.525
50 and above	−0.71 (−1.85, 0.43)	0.222	−0.98 (−2.25, 0.29)	0.131	−0.93 (−1.73, −0.12)	0.025^∗^

*Gender*						
Male	Reference					
Female	−0.14 (−0.52, 0.24)	0.472	−0.27 (−0.70, 0.15)	0.213	0.05 (−0.22, 0.33)	0.697

*Marital status*						
Married	Reference					
Single	0.35 (−0.06, 0.77)	0.098	0.29 (−0.17, 0.75)	0.214	0.01 (−0.29, 0.30)	0.957
Divorced	0.13 (−0.47, 0.74)	0.667	−0.02 (−0.69, 0.66)	0.960	−0.11 (−0.54, 0.32)	0.616

*Level of education*						
Diploma	Reference					
Bachelor’s degree	−0.24 (−0.66, 0.17)	0.249	−0.33 (−0.79, 0.13)	0.158	−0.05 (−0.34, 0.24)	0.744
Master’s/PhD degrees	−0.50 (−1.17, 0.16)	0.141	−0.49 (−1.22, 0.25)	0.199	−0.59 (−1.06, −0.12)	0.016^∗^

*Years of experience*						
1–5 years	Reference					
6–10 years	0.18 (−0.49, 0.86)	0.594	0.08 (−0.68, 0.83)	0.842	0.22 (−0.26, 0.70)	0.361
11–15 years	0.16 (−0.50, 0.81)	0.643	0.07 (−0.66, 0.80)	0.849	0.26 (−0.20, 0.73)	0.265
16–20 years	0.12 (−0.62, 0.87)	0.747	−0.07 (−0.89, 0.76)	0.878	0.26 (−0.27, 0.78)	0.341
More than 20 years	0.62 (−0.21, 1.46)	0.145	0.45 (−0.48, 1.38)	0.344	0.58 (−0.01, 1.17)	0.056

*Professional title*						
Staff nurse	Reference					
Charge nurse	0.61 (0.16, 1.06)	0.008^∗^	0.64 (0.14, 1.13)	0.013^∗^	0.20 (−0.12, 0.51)	0.228
Head nurse	0.23 (−0.40, 0.87)	0.473	0.26 (−0.44, 0.97)	0.466	−0.03 (−0.48, 0.42)	0.894
Nursing supervisor/coordinator/manager	0.36 (−0.25, 0.97)	0.250	0.25 (−0.42, 0.93)	0.465	0.36 (−0.08, 0.79)	0.107

^∗^Statistically significant.

### 4.5. Pearson Correlation Analyses Between MAW, WE, and PsyCap

Figure 2 illustrates the Pearson correlation between PsyCap, MAW, and WE. Panel A shows a moderate positive correlation between PsyCap and MAW (*r* = 0.67, *p* < 0.001), indicating that higher levels of PsyCap were associated with greater MAW. Similarly, Panel B demonstrates a moderate positive correlation between PsyCap and WE (*r* = 0.67, *p* < 0.001), indicating that higher levels of PsyCap were associated with greater WE. Panel C presents a strong positive correlation between MAW and WE (*r* = 0.87, *p* < 0.001), suggesting that increased MAW was strongly associated with higher levels of WE.

FIGURE 2Pearson correlation relationships between MAW, WE, and PsyCap.

**Figure:**
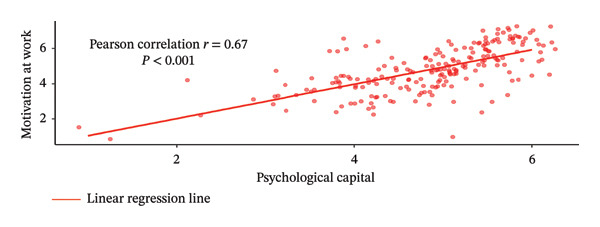
(a)

**Figure:**
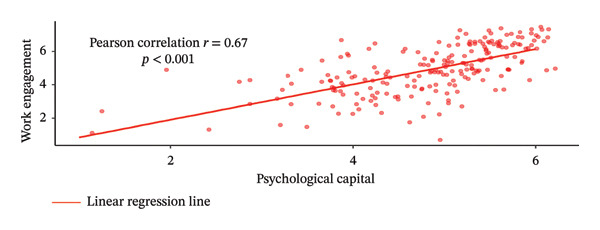
(b)

**Figure:**
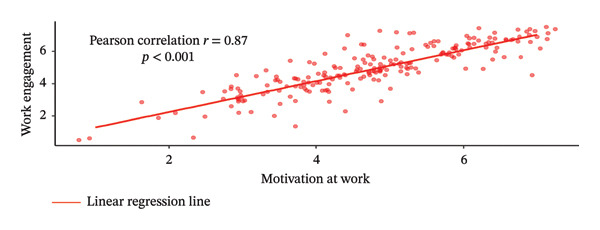
(c)

### 4.6. The Mediation Analyses

**FIGURE 3:**
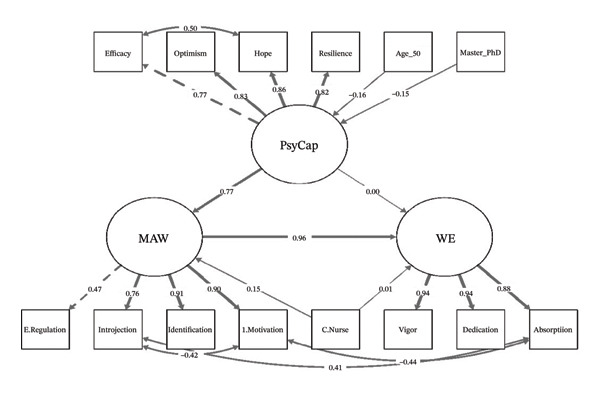
SEM showing the relationships between MAW, WE, and PsyCap. All paths are displayed in grayscale tones for accessibility, and the thickness of the arrow is related to standardized coefficient magnitude. Model fit indices indicate an acceptable fit (*χ* ^2^/df = 2.35, CFI = 0.96, TLI = 0.95, RMSEA = 0.08, SRMR = 0.04).

The indirect effect of PsyCap on WE through MAW was statistically significant (*ß* = 0.83, 95% CI: 0.69 to 0.97, *p* < 0.001), as well as the direct effect (*ß* = 0.23, 95% CI: 0.10 to 0.40, *p* < 0.001). Further, the total effect of PsyCap on WE was significant (*ß* = 1.06, 95% CI: 0.89 to 1.26, *p* < 0.001). The proportion of mediated effect was 0.78 (95% CI: 0.66 to 0.90, *p* < 0.001), indicating that a substantial portion of PsyCap influences on WE was mediated through MAW (Table 5). Although the bootstrapped mediation model (Table 5) showed a significant direct effect of PsyCap on WE (*ß* = 0.23, *p* < 0.001), indicating partial mediation, the SEM (Figure 3), which included latent variables and covariates, revealed a nonsignificant direct path (*ß* = 0.00, *p* = 0.938), supporting full mediation in the structural framework.

**TABLE 5:** Mediation analyses for the relationship between the total scores of PsyCap, MAW, and WE.

Effects	*ß* (95% CI)	*p*
The effect of PsyCap on WE through MAW		
Average causal mediation effect of PsyCap on WE through MAW (indirect effect)	0.83 (0.69, 0.97)	< 0.001^∗^
Average direct effect of PsyCap on WE	0.23 (0.10, 0.40)	< 0.001^∗^
Total effect of PsyCap on WE	1.06 (0.89, 1.26)	< 0.001^∗^
Proportion of mediated effect of PsyCap through MAW	0.78 (0.66, 0.90)	< 0.001^∗^

*Note: ß*: regression coefficient.

Abbreviation: CI, confidence Interval.

^∗^Statistically significant.

### 4.7. The SEM Between MAW, WE, and PsyCap

The measurement model showed that all observed variables significantly loaded on their respective latent constructs, with standardized coefficients ranging from 0.47 to 0.94 (*p* < 0.001), showing strong and reliable indicators for each latent variable.

In the structural model, PsyCap had a significant direct effect on MAW (*ß* = 0.77, *p* < 0.001) but not on WE directly (*ß* = 0.00,​ *p* = 0.938). However, MAW had a substantial direct effect on WE (*ß* = 0.96, *p* < 0.001), indicating that the influence of PsyCap on WE was fully mediated by MAW. The indirect effect of PsyCap on WE through MAW was significant (*ß* = 0.74, *p* < 0.001), accounting for most of the total effect (*ß* = 0.75, *p* < 0.001). The role of professional title was highlighted, with being a charge nurse significantly associated with higher MAW scores (*ß* = 0.15, *p* = 0.004). The indirect effect of charge nurse on WE through MAW was significant (*ß* = 0.15, *p* = 0.003), indicating that the influence of charge nurse status on WE was primarily mediated by MAW.

Demographic variables results showed that being 50 years or older (*ß* = −0.16, *p* = 0.031) and holding a master’s or PhD (*ß* = −0.15, *p* = 0.041) were negatively associated with PsyCap. The model fit indices suggested an acceptable fit (*χ* ^2^/df = 2.35, CFI = 0.96, TLI = 0.95, RMSEA = 0.08, SRMR = 0.04) (Figure 3). See Supporting Table S1.

## 5. Discussion

Most participants were female, 30 and 39 years old, and holding a bachelor’s degree, which reflects the common demographic profile reported among MHNs in Saudi Arabia. The finding showed that MHNs had a moderate level of motivation (*M* = 4.8 ± 1.3), which aligns with several studies indicating that motivation varies depending on a range of intrinsic and extrinsic factors in mental health settings [39, 40]. Based on JD‐R theory, job resources are crucial in enhancing employees’ motivation and engagement [26]. Additionally, SDT suggests that intrinsic motivation plays an important role in sustaining WE [27]. Therefore, tailored interventions among MHNs that provide adequate job resources and foster intrinsic motivation might be required to maintain and enhance motivation.

The findings also showed that WE levels were high (*M* = 4.9 ± 1.4), especially in the dedication subscale, which can be explained as MHNs demonstrate enthusiasm and commitment to their roles. This is consistent with previous findings [41]. Various levels of dedication and vigor have been reported in prior studies conducted during challenging periods [11, 13], showing that engagement can be a protective factor against occupational stress. According to JD‐R theory [26], job demands can lead to strain, while job resources foster engagement and buffer the impact of job stress.

In this study, results indicated a high overall PsyCap (*M* = 4.8 ± 0.9) across resilience, efficacy, hope, and optimism, which is crucial for personal and professional development. This finding supports JD‐R theory, as psychological resources have a role in motivating MHNs to be more engaged in their roles [26]. Across different studies, there is significant variability in PsyCap levels. For example, in Asia, nurses in intensive care units experience low PsyCap levels due to specific stressors and cultural factors [42]. Therefore, developing targeted interventions aimed at enhancing resilience and optimism among MHNs must consider the influence of the cultural and organizational context.

In this study, charge nurses had the highest levels of motivation and engagement compared to other, which may reflect the impact of leadership responsibilities on both intrinsic and extrinsic motivational factors [43, 44]. This is consistent with a previous study [45] that found that work position is a key factor contributing to high WE. The findings of the current study reported that nurses aged 50 and above exhibited lower PsyCap levels, consistent with previous research [46]. Additionally, the finding of a negative association between an advanced education and lower PsyCap may suggest that nurses are placed in positions where their advanced skills and higher education are not fully utilized, leading to a mismatch with their responsibilities. Such mismatch can diminish feelings of optimism and efficacy [43, 47]. From the JD‐R and SDT theoretical frameworks, a lack of job resources may reduce PsyCap and well‐being and were associated with lower nursing stress, suggesting that the work environment impacts the level of PsyCap and subjective well‐being [48]. Hence, optimizing nurse role alignment and providing opportunities for advanced degree nurses may strengthen their PsyCap.

The results of this study indicated that higher levels of PsyCap were associated with greater MAW among MHNs, consistent with previous studies [49]. Further, higher levels of PsyCap were associated with greater WE, consistent with findings across various settings [50–52]. This relationship can be explained by MHNs with high PsyCap, namely hope, optimism, self‐efficacy, and resilience, being more capable of sustaining motivation and enthusiasm despite workplace stressors. In line with SDT, PsyCap fulfills basic psychological needs, thus fostering intrinsic motivation and sustained engagement. Further, the results revealed that increased MAW was strongly associated with higher WE, consistent with previous research showing that MAW positively influences WE through both intrinsic and extrinsic motivational factors [53].

In the mediation analysis, PsyCap influenced WE both directly and indirectly through MAW. This means that motivation serves as a psychological mechanism linking PsyCap to WE. Previous studies support the significant influence of PsyCap on WE [54]. The study provides novel insights into the literature by clarifying the motivational pathway linking PsyCap and WE, consistent with the assumptions of the JD‐R and SDT frameworks. Therefore, supportive leadership and organizational practices are needed to implement strategies aimed at increasing motivation levels through strengthening PsyCap among MHNs to ensure their engagement.

### 5.1. Implications for Nursing

The results of this study showed the importance of enhancing MAW to improve WE among MHNs. Therefore, nurse managers and administrators should implement strategies that increase nurses’ motivation to improve WE and nurse retention. Healthcare organizations should consider creating policies that improve nurses’ motivation and apply training programs that focus on improving nurses’ PsyCap—through building hope, resilience, optimism, and self‐efficacy. Fostering a supportive environment in high‐demand settings such as psychiatric care is important to maintain engaged MHNs. These implications will improve MHNs’ well‐being and lead to better patient care outcomes.

### 5.2. Limitations and Recommendations for Future Study

The strength of this study includes a novel investigation of MAW as a mediator between PsyCap and WE among MHNs in Saudi Arabia, which adds context‐specific insights into MAW, PsyCap, and WE. The limitation of this study is the nature of the cross‐sectional design, which limits the ability to determine the causality between PsyCap, MAW, and WE. Reliance on self‐reported questionnaires may lead to response bias due to social desirability. Using a single hospital may introduce selection bias, as nurses may not represent all MHNs in Saudi Arabia. Further, the study was conducted in one geographic region, which limited the generalizability of findings to other geographic regions in Saudi Arabia. Therefore, a longitudinal study is needed to understand the relationship between MAW, WE, and PsyCap in MHNs. Further, a qualitative or mixed‐methods approach would add to the body of literature and gain deeper insights into the lived experience of MHNs regarding PsyCap, engagement, and motivation.

## 6. Conclusions

This study examined the relationship between PsyCap, MAW, and WE among MHNs, with a focus on the mediation role of MAW. The findings revealed positive associations between PsyCap, MAW, and WE, indicating that higher PsyCap levels are linked to greater motivation and engagement. MHNs who exhibit stronger motivation tend to demonstrate higher engagement in their roles.

The novelty of this study is identifying MAW as a mediator in the relationship between PsyCap and WE among MHNs. Thus, nurses with higher levels of hope, optimism, resilience, and self‐efficacy are more motivated, which in turn enhanced their vigor, dedication, and absorption at work. The study findings highlight the importance of developing targeted interventions to strengthen PsyCap and maintain motivation, thus fostering greater engagement, improving nurse well‐being, and promoting patient care outcomes.

**Nomenclature**
MHNs: Mental health nurses
PsyCap: Psychological capital
MAW: Motivation at work
WE: Work engagement
JD‐R: Job demands‐resources
SDT: Self‐determination theory
MDs: Mean differences
CIs: Confidence intervals
SEM: Structural equation modeling
MAWS: Motivation at Work Scale
PCQ‐12: Psychological Capital Questionnaire
UWES‐9: Utrecht Work Engagement Scale
RMSEA: Root mean square error of approximation
CFI: Comparative Fit Index
TLI: Tucker–Lewis Index
SRMR: Standardized root mean square residual

## Author Contributions

Samirh Said Alqhtani and Nouf Afit Aldhafeeri designed the study and supervised its progress and helped revise the manuscript and respond to the reviewer. Abdulraheem Mulfi Almutairy, Aryam Suhail Alotaibi, Arwa Alsharekh, Sarah Alanazi, Laila Al Soraia, and Norah Alharbi participated in data collection and manuscript writing. All researchers participated in the statistical analyses, discussion, implications, and recommendations.

## Funding

This study declares no specific grant received from any funding agency.

## Disclosure

All authors read and approved the published version of the manuscript.

## Conflicts of Interest

The authors declare no conflicts of interest.

## Supporting Information

Supporting Table 1 provides a summary of the structural equation model for the relationship between PsyCap, MAW, and WE.

## Supporting information


**Supporting Information** Additional supporting information can be found online in the Supporting Information section.

## Data Availability

The data that support the findings of this study are available from the corresponding author upon reasonable request.
