# Supplementary material for: Psychological Capital and Work Engagement Among Mental Health Nurses in Saudi Arabia: The Mediation Role of Motivation at Work
Source: J Nurs Manag. 2026 Apr 28;2026:6938783. doi: 10.1155/jonm/6938783 (PMC13123190; doi:10.1155/jonm/6938783)
Supplement: Supplementary file 1 — Supporting Information Additional supporting information can be found online in the Supporting Information section. [file JONM-2026-6938783-s001.docx]

| **Supplementary Table S1: Summary of the structural equation model for the relationship between Psychological Capital (PsyCap), Motivation at Work (MAW), and Work Engagement (WE)** | | | | |
| --- | --- | --- | --- | --- |
| **Model** | **Coefficient** | **SE** | **Std. Coefficient** | **p** |
| **Measurement models** |  |  |  |  |
| **PsyCap** |  |  |  |  |
| Efficacy | 1.00 | 0.00 | 0.77 | < 0.001* |
| Optimism | 1.10 | 0.09 | 0.83 | < 0.001* |
| Hope | 1.04 | 0.06 | 0.86 | < 0.001* |
| Resilience | 1.07 | 0.09 | 0.82 | < 0.001* |
| **CR** | 0.87 | | | |
| **AVE** | 0.67 | | | |
| **MAW** |  |  |  |  |
| External Regulation | 1.00 | 0.00 | 0.47 | < 0.001* |
| Introjection | 1.65 | 0.24 | 0.76 | < 0.001* |
| Identification | 1.87 | 0.25 | 0.91 | < 0.001* |
| Internal Motivation | 1.94 | 0.26 | 0.90 | < 0.001* |
| **CR** | 0.85 | | | |
| **AVE** | 0.61 | | | |
| **WE** |  |  |  |  |
| Vigour | 1.00 | 0.00 | 0.94 | < 0.001* |
| Dedication | 0.93 | 0.03 | 0.94 | < 0.001* |
| Absorption | 0.88 | 0.04 | 0.88 | < 0.001* |
| **CR** | 0.93 | | | |
| **AVE** | 0.85 | | | |
| **Structural model** |  |  |  |  |
| **Direct effects** |  |  |  |  |
| Age 50 or more on PsyCap | -0.60 | 0.28 | -0.16 | 0.031* |
| Master/PhD on PsyCap | -0.39 | 0.19 | -0.15 | 0.041* |
| MAW on WE (path b) | 1.90 | 0.28 | 0.96 | < 0.001* |
| PsyCap on WE (path c) | 0.01 | 0.11 | 0.00 | 0.938 |
| Charge Nurse on WE (path e) | 0.04 | 0.11 | 0.01 | 0.740 |
| Charge Nurse on MAW (path d) | 0.26 | 0.09 | 0.15 | 0.004* |
| PsyCap on MAW (path a) | 0.72 | 0.11 | 0.77 | < 0.001* |
| **Indirect effect and mediation** |  |  |  |  |
| Indirect effect of PsyCap on WE (a*b) | 1.37 | 0.16 | 0.74 | < 0.001* |
| Total effect of PsyCap on WE (c+ a*b) | 1.38 | 0.13 | 0.75 | < 0.001* |
| Indirect effect of charge nurse on WE (d*b) | 0.49 | 0.16 | 0.15 | 0.003* |
| Total effect of charge nurse on WE (e + d*b) | 0.53 | 0.17 | 0.16 | 0.002* |
| **Covariances** |  |  |  |  |
| Efficacy~~Hope | 0.16 | 0.04 | 0.50 | < 0.001* |
| Introjection~~Absorption | 0.29 | 0.06 | 0.41 | < 0.001* |
| Internal Motivation~~Absorption | -0.20 | 0.04 | -0.44 | < 0.001* |
| Introjection~~ Internal Motivation | -0.30 | 0.06 | -0.42 | < 0.001* |
| **Fit indices** |  |  |  |  |
| χ2/df | 2.35 | | | |
| CFI | 0.96 | | | |
| TLI | 0.95 | | | |
| RMSEA | 0.08 | | | |
| SRMR | 0.04 | | | |
